# Sex-Specific Association of Body Mass Index with Hippocampal Subfield Volume and Cognitive Function in Non-Demented Chinese Older Adults

**DOI:** 10.3390/brainsci14020170

**Published:** 2024-02-08

**Authors:** Shaohui Lin, Lijuan Jiang, Kai Wei, Junjie Yang, Xinyi Cao, Chunbo Li

**Affiliations:** 1 Shanghai Key Laboratory of Psychotic Disorders, Shanghai Mental Health Center, Shanghai Jiao Tong University School of Medicine, Shanghai 200030, China; shenhui1030@163.com (S.L.); ljjiang2012@163.com (L.J.); junjieyang@sjtu.edu.cn (J.Y.); 2 Department of Geriatrics, Shanghai Ninth People’s Hospital, Shanghai Jiao Tong University School of Medicine, Shanghai 200011, China; 3 Department of Traditional Chinese Medicine, Shanghai Mental Health Center, Shanghai Jiao Tong University School of Medicine, Shanghai 201108, China; wei.kai@hotmail.com; 4 Shanghai Institute of Traditional Chinese Medicine for Mental Health, Shanghai 201108, China; 5 Clinical Neurocognitive Research Center, Shanghai Mental Health Center, Shanghai Jiao Tong University School of Medicine, Shanghai 200030, China; 6 Institute of Psychology and Behavioral Science, Shanghai Jiao Tong University, Shanghai 200030, China; 7 CAS Center for Excellence in Brain Science and Intelligence Technology (CEBSIT), Chinese Academy of Sciences, Shanghai 200030, China

**Keywords:** body mass index, hippocampal subfield, cognitive function, sex difference, magnetic resonance imaging

## Abstract

Recent research suggests a possible association between midlife obesity and an increased risk of dementia in later life. However, the underlying mechanisms remain unclear. Little is known about the relationship between body mass index (BMI) and hippocampal subfield atrophy. In this study, we aimed to explore the associations between BMI and hippocampal subfield volumes and cognitive function in non-demented Chinese older adults. Hippocampal volumes were assessed using structural magnetic resonance imaging. Cognitive function was evaluated using the Repeatable Battery for the Assessment of Neuropsychological Status (RBANS). A total of 66 participants were included in the final analysis, with 35 females and 31 males. We observed a significant correlation between BMI and the hippocampal fissure volume in older females. In addition, there was a negative association between BMI and the RBANS total scale score, the coding score, and the story recall score, whereas no significant correlations were observed in older males. In conclusion, our findings revealed sex-specific associations between BMI and hippocampal subfield volumes and cognitive performance, providing valuable insights into the development of effective interventions for the early prevention of cognitive decline.

## 1. Introduction

With an increasingly aging population, dementia and cognitive decline have emerged as major public health concerns around the world, magnifying the resulting economic burden on individuals, families, and society [1]. However, there are currently no effective treatments available for dementia. Therefore, there is an urgent need to identify risk factors and investigate effective interventions that could potentially facilitate early prevention of cognitive decline.

In recent years, an increasing number of studies have linked obesity to cognitive decline, particularly in relation to Alzheimer’s disease (AD) [2,3,4,5,6,7]. Notably, a recent study identified midlife obesity as one of the primary modifiable factors strongly correlated with dementia [8]. Maintaining an overweight status (body mass index (BMI) ≥ 25 kg/m^2^) during midlife was found to increase the risk of cognitive impairment and dementia in later life [9]. Conversely, being overweight in the later stages of life may be associated with a reduced risk of dementia [8,10]. Nevertheless, the precise mechanisms underlying these associations remain unclear [11].

Brain imaging plays a crucial role in examining the mechanisms underlying structural and functional brain abnormalities that occur with aging and dementia. Recently, magnetic resonance imaging (MRI) has become a popular and rapidly advancing tool for investigating the neurobiology underlying variations in cognitive performance related to BMI in humans [12]. Several small-scale studies have found connections between BMI and changes in brain structure [13,14,15,16,17,18,19,20], including, specifically, alterations in the hippocampus [21]. However, there is less consistency in the associations between obesity and subcortical abnormalities [22,23,24,25,26], as well as variations in hippocampal changes across different studies [27,28]. Therefore, further research is needed to better understand the associations between BMI and subcortical regions, with a particular focus on the hippocampus.

During the aging process, the hippocampus is more vulnerable to atrophy than other brain regions [29]. Preserved cognitive function in older individuals has been associated with increased hippocampal volume, even in the presence of substantial AD pathology [30]. However, examining general morphometric changes in the whole hippocampus may not sufficiently elucidate specific neurological deterioration. It has been observed that hippocampal atrophy is more prevalent in certain subfields rather than in the total hippocampal volume, which may be indicative of future cognitive decline [30,31]. The hippocampus is a heterogeneous structure encompassing several interconnected and functionally specialized subfields. These include cornu ammonis (CA) areas 1–4, the granule cell layer of the dentate gyrus (GC-DG), the molecular layer, the subiculum, the fimbria, the hippocampal tail, and the hippocampal fissure. The hippocampal fissure serves as an interstitial space between the dentate gyrus and the subiculum, and its enlargement indicates early stages of hippocampal and medial temporal lobe atrophy [32,33]. Previous studies have demonstrated associations between hippocampal subfield volumes and cognitive function in older adults [34,35]. However, little is known about the correlations between BMI and hippocampal subfield atrophy in Chinese older adults.

Moreover, there are notable disparities in brain structure and cognitive function between males and females. Estrogen has significant and protective effects on cerebrovascular function [36]. Accelerated aging of the ovaries after menopause can cause metabolic dysfunction and cognitive deficits in female mice [37]. Gaining insights into the sex-specific behavior of brain structures during the normal aging process is crucial for tailoring individualized treatments [38]. However, little attention has been paid to the investigation of sex differences in hippocampal subfields in older adults. Ystad et al. [39] demonstrated that hippocampal volumes could serve as predictors of cognitive performance in older females. However, Pruessner et al. [40] and Li et al. [41] reported that age-related hippocampal atrophy was only observed in males. Therefore, further studies are needed to investigate the sex-specific associations between BMI, hippocampal subfield volumes, and cognitive function.

In the present study, we focused on the specific age group of 65–75 years old and used structural MRI to investigate the relationship between BMI and changes in hippocampal subfields, with the aim of revealing the associated sex differences in this relationship in non-demented, community-dwelling older adults.

## 2. Methods

### 2.1. Study Design and Participants

This study was approved by the human research ethics committees of Shanghai Mental Health Center (approval number: 2013-40, approval date: 12 June 2014) and Tongji Hospital, Shanghai (approval number: LL (H)-09-04, approval date: 20 February 2009), China. Participants were community-dwelling older adults who lived in neighborhoods located in the Jingan and Putuo Districts of Shanghai. They were recruited through posters, dispatched notices, and broadcasting by local neighborhood committees or service centers. Before participation, written informed consent was obtained from all participants. Inclusion criteria were as follows: individuals aged 65–75 years with more than one year of formal education living independently in the community; no disabilities; no visual, hearing, or communication impairments; no severe psychotic disorders or physical illnesses; and achieving a score of 19 or higher on the Chinese version of the Mini-Mental State Examination (CMMSE) for primary education, or a score of 24 or higher for middle school education and above. It is important to note that the standard cut-off point for CMMSE scores is lower in China due to the comparatively lower level of education [42]. Exclusion criteria included severe cognitive decline; diagnosis of Alzheimer’s disease; major neurological and/or psychiatric disorders, such as brain cancer, cerebral infarction, cerebral hemorrhage, malnutrition, major depressive disorder, or schizophrenia; and history of brain trauma or surgery. BMI was determined based on height and weight records, calculated as weight in kilograms divided by height in meters squared (kg/m^2^).

### 2.2. Cognitive Measurement

Cognitive function was assessed in each participant using the Repeatable Battery for the Assessment of Neuropsychological Status (RBANS) [43], which has demonstrated good validity and reliability in Chinese community-dwelling older adults [44]. The RBANS consists of 12 subtests that produce 5 index scores for the following cognitive domains: immediate memory (list learning and story memory), visuospatial/constructional (figure copying and line orientation), language (picture naming and semantic fluency), attention (digit span and coding), and delayed memory (list recall, list recognition, story recall, and figure recall), in addition to an overall cognitive function score. Global cognition at enrollment was assessed using the Chinese version of the Mini-Mental State Examination (CMMSE), which has been shown to have good validity and reliability in the Chinese population [45]. All cognitive assessments were administered by trained research assistants following the protocol described in the manual.

### 2.3. MRI Acquisition

All participants underwent imaging on a 3.0 Tesla scanner (Siemens Medical, Erlangen, Germany) with a standard 12-channel head coil using a consistent structural scanning protocol. High-resolution T1-weighted imaging was performed in the sagittal plane using a fast, three-dimensional, gradient-spoiled gradient echo sequence with the following parameters: repetition time, 1900 ms; echo time, 3.43 ms; flip angle, 9°; matrix size, 256 × 256; field of view, 240 × 240 mm^2^; slice thickness, 1 mm; voxel size, 0.9 × 0.9 × 1.0 mm^3^; and 160 slices. Each scan took 5 min. Images were reconstructed and visually inspected for major artifacts, including motion, ringing, wrap-around, and neurological abnormalities, before further processing. MRI data processing was performed by a research technician who was blinded to all clinical information.

### 2.4. Image Preprocessing

The entire hippocampal formation was segmented using the standard FreeSurfer segmentation procedure in the FreeSurfer 6.0 image analysis suite (http://surfer.nmr.mgh.harvard.edu/, accessed on 23 January 2017) [46]. The processing flow included the following steps: motion correction averaging of the two T1-weighted volumes, removal of non-brain tissue using a hybrid watershed/surface deformation procedure [47], automated transformation into the Talairach reference space, and segmentation of subcortical white matter and gray matter structures using a probabilistic brain atlas [46]. In addition, hippocampal subfields were automatically segmented using FreeSurfer 6.0. Following the previous methodology [48], the following 13 subfield volumes were calculated on each side of the hippocampus: CA1, CA2/3, CA4, GC-DG, fimbria, subiculum, pre-subiculum, para-subiculum, molecular layer, hippocampal amygdala transition area (HATA), hippocampal tail, hippocampal fissure, and the whole hippocampus. The segmentation results were visually inspected for errors in all datasets without manual editing. The volumes of the hippocampal subfields and the whole hippocampus are the sum of the left and right hippocampi. To account for differences in head size, we calculated the estimated total intracranial volume (eTIV) to adjust the subfield volumes in the subsequent statistical analysis.

### 2.5. Statistical Analyses

All statistical analyses were performed using IBM SPSS Statistics version 22 (IBM Corporation, Somers, NY, USA). The statistical significance level was set at *p* < 0.05. Categorical and continuous variables are presented as numbers and means (standard deviations), respectively. The Mann–Whitney test and Student’s *t*-test were used to compare variables, such as age, education, and CMMSE score, between females and males. Partial correlation analysis was used to examine the potential association between BMI, hippocampal subfield volumes, and cognitive performance. Additionally, we stratified the total sample by sex and repeated the above models in males and females separately. We then used multivariate linear regression models to further examine the significant associations between BMI and hippocampal subfield volumes and cognitive scores, adjusting for confounders, such as age, sex, and education. Regression coefficients with 95% confidence intervals (β (95% CI)) and the adjusted model fit (adjusted *R*^2^) were calculated. False discovery rate (FDR)-corrected *p*-values were calculated to account for multiple comparisons, as described in previous studies [49].

## 3. Results

### 3.1. Demographic and Clinical Characteristics of Participants

**Figure 1:**
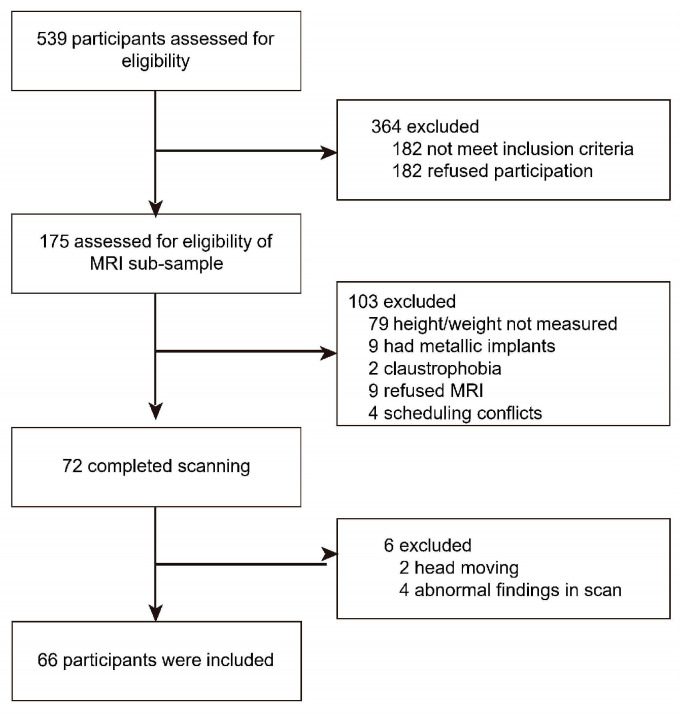
Participant enrollment flow chart.

The participant enrollment flowcharts are presented in Figure 1. Out of the initial 539 individuals contacted for participation between November 2013 and September 2014, a total of 175 individuals were included in the MRI subsample. Of these, 103 participants were excluded for a variety of reasons; of these, 79 did not provide height and weight measurements, 9 had metal implants, 2 reported claustrophobia, 9 declined the MRI scan, and 4 had scheduling conflicts. As a result, a final sample of 72 participants met the eligibility criteria and completed the MRI scans. However, a further 6 participants had to be excluded due to head movement (*n* = 2) and abnormal scan findings (*n* = 4). Thus, after excluding a total of 109 individuals, the final analysis included 66 participants in the present study (Figure 1).

The demographic and clinical characteristics of the participants are presented in Table 1. Of the 66 individuals included in this study, 35 were female and 31 were male. The participants had a mean age of 68.96 (3.04) years and a mean CMMSE score of 28.26 (1.34) points. The mean BMI of the entire sample was 23.44 (3.291), ranging from 16.98 to 32.28. There were no significant differences observed between females and males in terms of age, years of education, CMMSE score, or RBANS total scale score. However, it is important to note that the eTIV volumes were significantly lower in females compared to males (Table 1).

### 3.2. Association between BMI and Hippocampal Subfield Volumes

**Figure 2:**
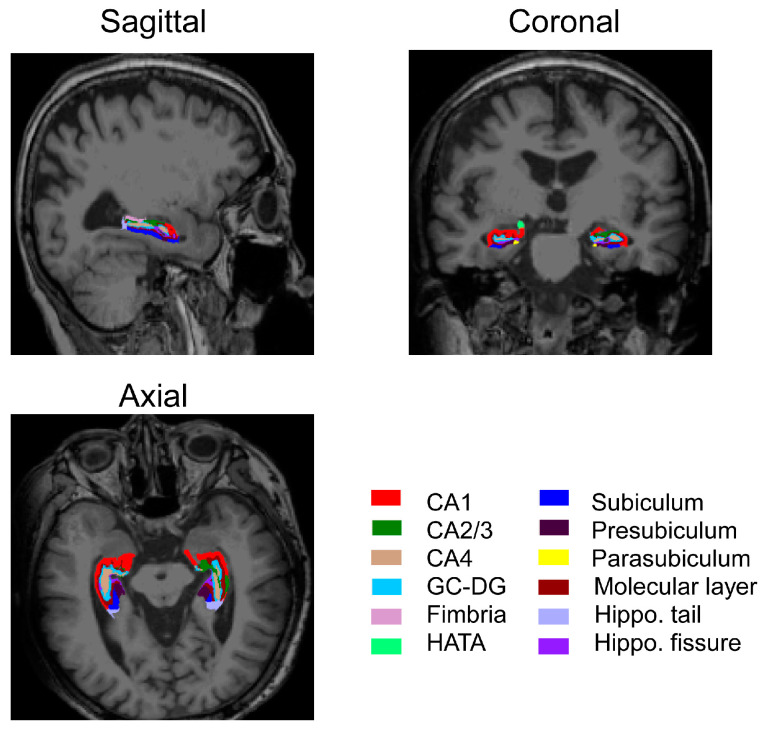
Automated segmentation of the hippocampal subfields. CA: cornu ammonis area, GC-DG: granule cell layer of the dentate gyrus, HATA: hippocampus–amygdala transition area.

**Figure 3:**
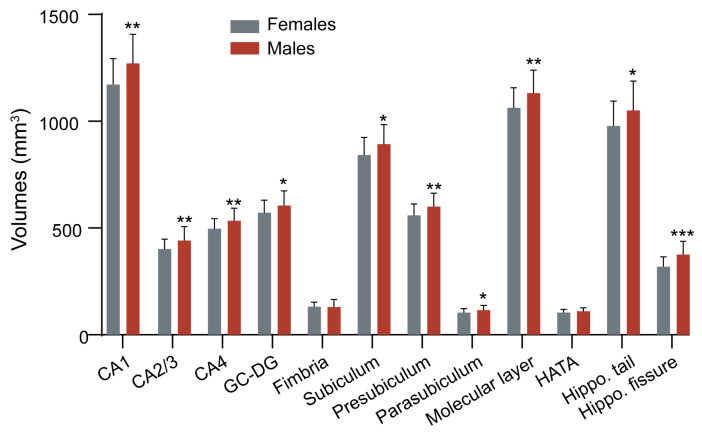
Comparisons of the volumes (mm^3^) of the hippocampal subfields between females and males from the cross-sectional sample. Bar graphs present the distributions of the mean hippocampal subfield volumes among participants. Error bars represent the standard deviation. * *p* < 0.05, ** *p* < 0.01, *** *p* < 0.001. Abbreviations: CA: cornu ammonis area, GC-DG: granule cell layer of the dentate gyrus, HATA: hippocampus–amygdala transition area.

The associations between BMI and volumes of hippocampal subfields were examined through partial correlation analysis and multivariate linear regression models. The hippocampal segmentations are shown in Figure 2. The statistical results of volumetric differences between the females and males are presented in Figure 3.

**Figure 4:**
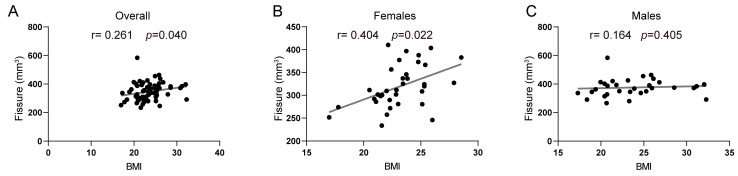
Correlations between BMI and hippocampal fissure volumes in the overall sample (**A**), females (**B**), and males (**C**).

As shown in Table 2, when adjusting for age, sex, education, and eTIV, BMI displayed a significant relationship with a specific subfield, the hippocampal fissure (*r* = 0.261, *p* = 0.040) (Figure 4A), in the overall sample. Given the previously reported sex differences in hippocampal structures [48,50,51], we further stratified the analysis by sex. Interestingly, the association between BMI and hippocampal fissure was only significant in older females (*r* = 0.404, *p* = 0.022) (Figure 4B), while it was not significant in males (*r* = 0.164, *p* = 0.405) (Figure 4C). To further investigate this significant relationship, we employed multivariate linear regression models in females, both unadjusted and adjusted for potential confounders. Supplementary Table S1 presents the results, indicating that after adjusting for age, sex, education, and eTIV, BMI showed a positive association with hippocampal fissures in both the total sample (*β* = 4.559 (0.209, 8.908), adjusted *R*^2^ = 0.253) and in females specifically (*β* = 7.732 (1.206, 14.258), adjusted *R*^2^ = 0.173). However, these findings did not survive correction for multiple comparisons (FDR-corrected *p* > 0.050) in our exploratory analyses. This could potentially be attributed to the relatively small sample size and limited power to detect underlying associations in this study.

### 3.3. Association between BMI and Cognitive Functions

**Figure 5:**
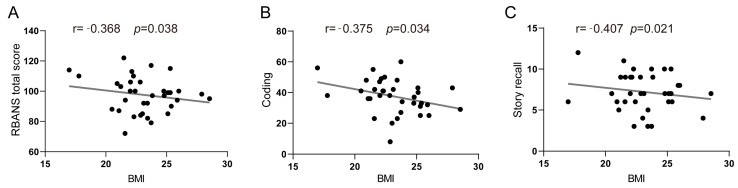
Correlations of BMI with the RBANS total scale score (**A**), coding (**B**), and story recall (**C**) in females.

Partial correlation analysis and multivariate linear regression models were implemented to investigate the associations between BMI and specific cognitive domains, as evaluated using the RBANS. As shown in Table 3, among older females, BMI exhibited a negative association with the RBANS total scale score (*r* = −0.368, *p* = 0.038) (Figure 5A), as well as with two subtest scores: coding (*r* = −0.375, *p* = 0.034) (Figure 5B) and story recall (*r* = −0.407, *p* = 0.021) (Figure 5C). In contrast, there were no significant associations observed between BMI and other cognitive domains, such as immediate memory, visuospatial/constructional ability, and language, following adjustment (*p* > 0.050). Furthermore, no notable correlations were found between BMI and RBANS domain index scores in older males (*p* > 0.050). In the multivariate linear regression models (see Supplementary Table S2), after controlling for potential confounders, BMI was found to be inversely associated with the RBANS total scale score (*β* = −1.741 (−3.380, −1.101), adjusted *R*^2^ = 0.244), coding (*β* = −0.367 (−0.674, −0.060), adjusted *R*^2^ = 0.320), and story recall (*β* = −1.794 (−3.447, −0.141), adjusted *R*^2^ = 0.131) scores. However, the associations between BMI and RBANS scores did not demonstrate statistical significance in multiple FDR-corrected comparison tests (corrected *p* > 0.05).

## 4. Discussion

In this cross-sectional study, we examined the associations of BMI with hippocampal subfield volume and cognitive function in non-demented, community-dwelling older adults in China. Our results showed sex-specific associations between BMI and these measures. Specifically, we observed a significant association between BMI and hippocampal fissure volume in older females. We also found a significant relationship between BMI and the RBANS total scale score and two subscale scores, coding and story recall. However, we did not observe any significant correlations between BMI and specific volumes of hippocampal subfields or cognitive scores in older males. These results suggest that a high BMI has a greater effect on hippocampal subfields and cognitive parameters in females than in males.

We observed an association between BMI and specific hippocampal subregions as well as cognitive performance. This association was found to be more significant in females than in males. Previous research studies have also supported these differences [3,17,52,53], indicating that the relationship between BMI and brain structure and cognition varies between sexes. Previous studies have shown that hippocampal volume predicts cognitive performance in older females but not in males [39]. Additionally, obesity independently increases the risk of cognitive impairment [4,54], with a greater effect in females [55,56,57]. There are several potential factors that could explain this sex-specific association. Firstly, the metabolic effects of sex steroid hormones during aging could lead to the production of different metabolites, resulting in age-related brain changes in females at an earlier stage than in males. There are sex hormone receptors, such as androgen receptors, estrogen receptors, and G-coupled protein receptor, in the hippocampus [58]. The expression of estrogen receptors in the hippocampus during aging was higher in older females than in males [59]. Sex differences in the hippocampus, at least in part, relate to steroid hormone manipulations [60]. In addition, differences in fat distribution and body composition between the sexes could explain the discrepant effects of BMI on the hippocampal subfield in older adults [57], creating a more vulnerable environment in females than in males. Genetic factors may also contribute to sex differences in brain structure and cognition [61]. During the development and progression of AD, a sex-specific genetic architecture may emerge with substantial APOE contributions to AD in females, including the sex-biased association between APOE and tau in amyloid-positive subjects [62,63,64]. These findings might suggest a potential opportunity to prevent cognitive decline in females. Timely interventions during this window may help to halt the progression of metabolic deficits and ultimately reduce the risk of dementia in females.

Notably, our findings revealed a significant association between higher BMI and larger hippocampal fissure volumes in non-demented older females. The hippocampal fissure possesses a higher fractional vascular density compared to other subfields of the hippocampus [65], and it is a susceptible subregion for Alzheimer’s disease pathology [66,67]. Enlargement of the hippocampal fissure is indicative of early gray matter atrophy within the hippocampal formation [68,69]. In Alzheimer’s disease, the hippocampal fissure has been identified as the sole hippocampal subfield exhibiting increased volume [70]. We found an association of BMI with hippocampal fissure volume, which has not been reported before. Previous studies have shown that a larger hippocampal fissure correlates with an increased risk of dementia [71] and may serve as a specific indicator of the conversion from MCI to AD [71]. Additionally, strong associations have been documented between the hippocampal fissure and polygenic Alzheimer’s disease risk scores [72]. Hibar DP et al. found that among the top variants in hippocampal subfield volume, Rs61921502 showed the largest effect in the right hippocampal fissure and a strong lateralized effect across right hippocampal subfields [73]. To sum up, BMI, accompanied by enlarged hippocampal fissures, may be one of the early indicators of cognitive impairment in non-demented older individuals, especially in females. However, we did not find significant associations between BMI and total hippocampal volume, as reported in previous studies [74,75]. This discrepancy may be due to the fact that our study participants comprised cognitively normal older individuals without significant hippocampal atrophy. 

Consistent with previous research suggesting that being overweight is associated with cognitive decline linked to a decline in cognitive performance among young and middle-aged adults [76,77], the present results extended the existing findings to older age groups. The RBANS total scale score, as well as the delayed memory index and immediate memory index, have been reported to be correlated with hippocampal and global brain capacity in older individuals [78,79]. In addition, brain amyloid plaque density and deposition, which are indicative of AD brain pathology, were significantly related to the RBANS total scale score and all five index scores, with higher scores being associated with less brain amyloid [80]. Our study showed a significant correlation between BMI and cognitive performance in a sample of non-demented, community-dwelling older adults. A systematic review also supports our findings, revealing a negative association between overweight and the risk of cognitive impairment. BMI also negatively affects the cognitive performance of fibromyalgia patients [81]. A cohort study from the Chinese Longitudinal Healthy Longevity Survey (CLHLS) supports the positive associations of overweight status with cognitive function in older adults, particularly in those with a higher plant-based diet index [10]. It should be pointed out that other factors, such as physical activity, may also play a role in modifying the association between BMI and cognition in the elderly. Such factors may amplify the effect of BMI on cognition. However, it is unclear to what extent these factors play a role in brain atrophy and cognitive decline. It is also important to note that differences in study populations and methodologies may contribute to inconsistent results between studies [82,83]. In addition, it should be considered that the association between BMI and cognitive dysfunction may differ between individuals from different racial or ethnic backgrounds [84].

The strength of our study is that we identified the relationship between BMI and a specific subfield of the hippocampus for the first time. To the best of our knowledge, this is the initial investigation to examine the association between BMI and volumes of hippocampal subfields in Chinese community-dwelling older adults. However, our study had several limitations. Firstly, the cross-sectional design of this clinical study could only reveal the association between BMI and volumes of hippocampal subfields as well as specific cognitive domains, but it could not establish a causal relationship between them. Secondly, our data were collected at a single community site, which may limit the generalizability of the study findings. Additionally, the small sample size may have led to a lack of statistical power to detect significant associations and appropriate effect sizes. Therefore, future longitudinal studies with larger sample sizes conducted in different communities are needed to validate our findings. Future research efforts should consider how modifiable risk factors, such as BMI, relate to imaging markers of cerebrovascular pathology and the extent to which they relate to clinical and cognitive outcomes. In addition, further research and longitudinal studies are warranted to explore whether weight control mitigates the risk of hippocampal atrophy in older adults.

## 5. Conclusions

Our findings exclusively revealed a significant association between BMI, hippocampal fissure, and cognitive performance in older Chinese females, while no such correlation was observed in males. Future longitudinal studies should examine the contribution of BMI to Aβ and tau accumulation, as this will provide additional insight into the extent to which modifiable risk factors contribute to the development of cerebrovascular pathology, as well as a deeper understanding of the mechanisms underlying the sex-specific correlations of BMI with the hippocampus. These findings may help to develop effective sex-stratified preventive and therapeutic interventions to promote hippocampal health and prevent cognitive decline at an early stage.

## Figures and Tables

**Table 1:** Clinical characteristics of study participants.

Characteristics	Entire Sample (*n* = 66)	Females (*n* = 35)	Males (*n* = 31)	*p* ^a^
Age (year)	68.90 ± 3.04	68.76 ± 2.96	69.06 ± 3.17	0.687
Education (year)	12.20 ± 2.80	12.09 ± 2.79	12.32 ± 2.84	0.864
BMI (kg/m^2^)	23.44 ± 3.291	23.16 ± 2.40	23.77 ± 4.09	0.467
CMMSE	28.26 ± 1.34	28.43 ± 1.24	28.06 ± 1.44	0.658
RBANS total scale score	95.636 ± 10.98	97.51 ± 11.71	93.52 ± 9.85	0.141
Immediate memory	86.35 ± 13.18	87.83 ± 12.67	84.68 ±13.75	0.336
Visuospatial/				
constructional ability	102.80 ± 15.96	102.46 ± 16.42	103.19 ± 15.69	0.853
Language	97.38 ± 8.54	97.74 ± 9.66	96.97 ± 7.20	0.540
Attention	101.48 ± 13.09	104.06 ± 14.21	98.58 ± 11.23	0.090
Delayed memory	97.59 ± 12.22	99.97 ± 11.63	94.90 ± 12.50	0.197
Whole hippocampus volume (mm^3^)	6642.11 ± 617.06	6425.65 ± 536.95	6886.51 ± 617.93	0.002 *
eTIV (cm^3^)	1487.84 ± 163.39	1378.04 ± 96.94	1611.81 ± 131.32	0.000 *

BMI, body mass index; eTIV, estimated total intracranial volume; CMMSE, Chinese version of the Mini-Mental State Examination; RBANS, Repeatable Battery for the Assessment of Neuropsychological Status. Data are presented as the mean ± standard deviation. ^a^ The *p*-value was based on the Student’s *t*-test or Mann–Whitney U test, as appropriate. * *p* < 0.05

**Table 2:** Partial correlation analysis of BMI with hippocampal subfield volumes.

Hippocampal Subfields	Overall (*n* = 66) ^a^		Males (*n* = 31) ^b^		Females (*n* = 35) ^b^	
	*r*	*p*	*r*	*p*	*r*	*p*
CA1	0.051	0.694	−0.096	0.629	0.286	0.112
CA2/3	0.166	0.197	0.108	0.585	0.264	0.144
CA4	0.016	0.899	−0.132	0.505	0.249	0.170
GC–DG	−0.051	0.692	−0.229	0.240	0.183	0.315
Fimbria	−0.167	0.195	−0.081	0.680	−0.314	0.080
Subiculum	−0.063	0.626	−0.216	0.269	0.200	0.272
Presubiculum	−0.053	0.682	−0.032	0.870	0.029	0.877
Parasubiculm	−0.048	0.712	−0.041	0.836	−0.113	0.537
Molecular layer	−0.004	0.976	−0.158	0.423	0.263	0.146
HATA	−0.086	0.506	−0.156	0.428	−0.041	0.823
Hippocampal tail	0.043	0.741	−0.046	0.815	0.296	0.100
Hippocampal fissure	0.261	0.040 *	0.164	0.405	0.404	0.022 *
Whole hippocampus	0.004	0.974	−0.136	0.489	0.256	0.157

CA, cornu ammonis; GC-DG, granule cell layer of the dentate gyrus; HATA, hippocampus–amygdala transition area; eTIV: estimated total intracranial volume. ^a^ Includes age, sex, education, and eTIV as covariates. ^b^ Includes age, education, and eTIV as covariates. The *p*-values with “*” indicate a significant correlation.

**Table 3:** Partial correlation analysis of BMI with cognitive functions measured using RBANS.

Cognitive Function	Overall (*n* = 66) ^a^		Males (*n* = 31) ^b^		Females (*n* = 35) ^b^	
	*r*	*p*	*r*	*p*	*r*	*p*
Immediate memory	0.049	0.708	0.214	0.273	−0.186	0.308
List learning	0.056	0.667	0.144	0.465	−0.030	0.869
Story memory	−0.025	0.849	0.147	0.454	−0.277	0.124
Visuospatial/						
constructional ability	−0.139	0.282	−0.012	0.951	−0.329	0.066
Figure copy	−0.204	0.112	−0.206	0.293	−0.094	0.611
Line orientation	0.002	0.988	0.124	0.528	−0.300	0.095
Language	0.117	0.364	0.227	0.245	−0.071	0.700
Picture naming	0.069	0.597	0.211	0.282	−0.007	0.972
Semantic fluency	0.169	0.188	0.221	0.258	−0.053	0.775
Attention	−0.123	0.340	0.140	0.477	−0.252	0.163
Digit span	−0.106	0.412	−0.045	0.821	0.034	0.854
Coding	−0.082	0.526	0.244	0.212	−0.375	0.034 *
Delayed memory	−0.013	0.921	0.129	0.514	−0.178	0.330
List recall	−0.009	0.944	0.023	0.906	−0.056	0.759
List recognition	−0.050	0.699	0.139	0.481	−0.320	0.074
Story recall	0.019	0.881	0.166	0.400	−0.407	0.021 *
Figure recall	0.126	0.330	0.176	0.369	0.003	0.989
RBANS total scale score	−0.056	0.665	0.252	0.196	−0.368	0.038 *

^a^ Includes age, sex, education, and CMMSE as covariates. ^b^ Includes age, education, and CMMSE as covariates. The *p*-values with “*” indicate a significant correlation.

## Data Availability

The raw data supporting the conclusions of this article will be made available upon request. The data are not publicly available due to patient privacy protection purposes.

## Supplementary Materials

The following supporting information can be downloaded at: https://www.mdpi.com/article/10.3390/brainsci14020170/s1, Table S1. Multivariate regression models assessing the association between BMI and hippocampal subfield volumes. Table S2. Multivariate regression models assessing the association between BMI and cognitive functions in females.
