# Supplementary material for: Sex-Specific Association of Body Mass Index with Hippocampal Subfield Volume and Cognitive Function in Non-Demented Chinese Older Adults
Source: Brain Sci. 2024 Feb 8;14(2):170. doi: 10.3390/brainsci14020170 (PMC10887390; doi:10.3390/brainsci14020170)
Supplement: Supplementary file 1 [file brainsci-14-00170-s001.zip › brainsci-2830087-supplementary.pdf]

Supplementary Material

Supplementary Table S1. Multivariate regression models assessing the association between BMI and hippocampal subfield volumes.

Model	Hippocampal subfields	*β* (95% CI)	*t*	*p*	Adjusted R^2 (*p*-value)
Overall-unadjusted[1]	Fissure	4.210 (−0.326, 8.747)	1.854	0.068	0.036 (0.068)
Female-unadjusted[1]	Fissure	9.100 (2.994, 15.206)	3.032	***0.005****	0.194 (0.005)
Overall-adjusted[2]	Fissure	4.559 (0.209, 8.908)	2.097	***0.040****	0.253 (0.000)
Female-adjusted[2]	Fissure	7.732 (1.206, 14.258)	2.420	***0.022****	0.173 (0.045)

Model 1 unadjusted

Model 2 includes age, education, and eTIV as covariates.

The *p*-values with "*" indicate a significant correlation.

Supplementary Table S2 Multivariate regression models assessing the association between BMI and cognitive functions in females.

Model	Cognitive functions	*β* (95% CI)	*t*	*p*	Adjusted R^2 (*p*-value)
Female-unadjusted[1]	Coding	−1.505 (−3.038,0.029)	−1.996	0.054	0.081 (0.054)
	Story recall	−0.162 (−0.498,0.174)	−0.983	0.333	−0.001 (0.333)
	RBANS total score	−0.936 (−2.631,0.758)	−1125	0.269	0.008 (0.269)
Female-adjusted[2]	Coding	−0.367 (−0.674, −0.060)	−2.438	***0.021****	0.320 (0.003)
	Story recall	−1.794 (−3.447, −0.141)	−2.216	***0.034****	0.131 (0.083)
	RBANS total score	−1.741 (−3.380, −1.101)	−2.168	***0.038****	0.244 (0.014)

Model 1 unadjusted

Model 2 includes age, education, and CMMSE as covariates.

The *p*-values with "*" indicate a significant correlation.
